# Estimation of Wideband Multi-Component Phasors Considering Signal Damping †

**DOI:** 10.3390/s23167071

**Published:** 2023-08-10

**Authors:** Dongfang Zhao, Shisong Li, Fuping Wang, Wei Zhao, Songling Huang

**Affiliations:** 1 Department of Electrical Engineering, Tsinghua University, Beijing 100084, China; dongfangthu@outlook.com (D.Z.); shisongli@tsinghua.edu.cn (S.L.); zhaowei@mail.tsinghua.edu.cn (W.Z.); huangsling@mail.tsinghua.edu.cn (S.H.); 2 China Electric Power Planning & Engineering Institute, Beijing 100120, China

**Keywords:** damping factor, least squares, matrix pencil, phasor measurement unit, singular value decomposition, wideband multi-component signal

## Abstract

Harmonic and interharmonic content in power system signals is increasing with the development of renewable energy generation and power electronic devices. These multiple signal components can seriously degrade power quality, trip thermal generators, cause oscillations, and threaten system stability, especially the interharmonic tones with positive damping factors. The first step to mitigate these adverse effects is to accurately and quickly monitor signal features, including frequency, damping factor, amplitude, and phase. This paper proposes a concise and robust index to identify the number of modes present in the signal using the singular values of the Hankel matrix and discusses the scope of its application by testing the influence of various factors. Next, the simplified matrix pencil theory is employed to estimate the signal component frequency and damping factor. Then their estimates are considered in the modified least-squares algorithm to extract the wideband multi-component phasors accurately. Finally, this paper designs a series of scenarios considering varying signal frequency, damping factor, amplitude, and phase to test the proposed algorithm thoroughly. The results verify that the proposed method can achieve a maximum total vector error of less than 1.5%, which is more accurate than existing phasor estimators in various signal environments. The high accuracy of the proposed method is because it considers both the estimation of the frequency number and the effect of signal damping.

## 1. Introduction

### 1.1. Background and Motivations

The diffusion of renewable energy generation has promoted the widespread use of power electronic devices, but also introduced harmonic and interharmonic tones into the power system signal and resulted in oscillation events. For example, 20 to 30 Hz oscillations with a non-zero damping factor were found in a radial connection between wind turbines and series capacitors [1], and 26.3 Hz oscillations with positive damping were captured in a grid-connected permanent magnet synchronous generator [2]. Higher-frequency oscillations, e.g., 250–350 Hz oscillations, were observed in the BorWin1 offshore wind farm in Germany [3], and interharmonics in the range of about 1.7–2.0 kHz were also found in several grid-connected inverters [4]. As a result, the difficulty of measuring phasors under power system signals over a wide frequency range of 10–2000 Hz with multi-tone and non-zero damping factors increases dramatically [5].

In addition to causing oscillations, the multiple harmonic and interharmonic tones can also degrade power quality, trip thermal power generators, and damage armatures and insulators [6,7]. The accurate estimation of these signal component characteristics is a key point to mitigate these negative effects effectively [6]. In addition, the accurate estimation of wideband multi-component phasors is beneficial for developing co-simulations of large power systems [8], for obtaining accurate distribution topology [9], for identifying sub-synchronous control interaction sources [10], etc. To conclude, the accurate measurement of these signal component characteristics is vital to ensure the stable operation of power systems.

### 1.2. Literature Review

Wideband multi-component phasors contain fundamental, harmonic, and interharmonic tones according to different frequencies. Among these phasors, the study of algorithms for measuring only the fundamental phasor is the most mature, including the international standard [11] and many estimators based on discrete Fourier transform (DFT) [12,13,14,15,16,17], least squares (LS) [18,19,20,21,22,23,24], etc. By extending the signal model, these fundamental phasor estimators can be developed to measure harmonic phasors, such as [25,26,27,28,29,30,31,32,33]. However, with the increase in interharmonic tones, the estimation error will significantly increase when using these algorithms to extract fundamental or harmonic phasors from signal samples. In contrast, the measurement of interharmonic phasors is more difficult due to the randomness of the number and frequency, and the non-zero damping factor of the tones in the measured signal.

There are some algorithms that try to estimate interharmonic parameters from phasor measurement unit (PMU) data. For example, classical fast Fourier transform (FFT) has been employed to analyze spectral PMU data in the second-level time window, and to obtain the amplitude of sub-/super-synchronous oscillations [34]. An interpolated DFT using 200 nominal cycles of synchrophasor data was applied to obtain the sub-synchronous frequency, damping factor, and amplitude [35]. An entropy function based on the upper and lower envelopes of PMU data was maximized to determine whether there was a 5–45 Hz oscillation. For data that may contain interharmonics, DFT analysis was applied to calculate their amplitudes using a window length of several seconds [36]. However, these algorithms typically tend to have a long response latency, and cannot estimate the interharmonic phase and high-frequency oscillation parameters. In addition, the interharmonic energy in PMU data is attenuated by synchrophasor algorithms [37].

Recently, another idea has been proposed to extract wideband multi-component phasors directly from sampled signals. In such cases, it is necessary to accurately identify the number and frequency of the modes in the measured signal. By improving the frequency resolution of DFT to 1 Hz [37,38], some methods detected harmonics above 5% of the fundamental amplitude and extracted the fundamental and harmonic phasors by infinite impulse response filters. Then, oscillation phasors in the range of 0–100 Hz were calculated based on the fundamental phasor. In addition, DFT and the least-squares algorithm were combined to realize multi-tone spectrum separation and measurement under a ten-nominal-cycle window [39]. The matrix pencil was used in the Hankel matrix [40] or the autocorrelation matrix [41,42,43] to obtain the number and frequency of different components. With the estimated component frequencies, the bandpass filter banks were designed by the LS algorithm to obtain the amplitude and phase of all components [44]. These methods have the merits of high estimation accuracy and a short time window. However, the reliability of the application requires further investigation, as the performance may be affected by whether the number of modes in the signal has been sufficiently identified. In addition, most of the above methods do not consider the effect of non-zero attenuation. As a result, the estimation accuracy of these algorithms may be degraded in complex signal environments.

### 1.3. Summary of Contributions

The critical point for accurate wideband multi-component phasor estimation is to reduce the influence of random signal component number and non-zero damping oscillations. Then, this paper proposes a wideband multi-component phasor estimator (WMPE). The proposal mainly includes presenting a concise and robust index to identify the signal component number, using the matrix pencil theory to obtain the signal component frequency and damping factor, and finally applying a modified LS algorithm that considers signal damping to estimate the phasors accurately. Compared to other existing works, the main contributions of this paper include the following.

(1) A concise index and criterion for identifying the number of modes in the measured signal with a non-zero damping factor is proposed. It is found to be more robust than the existing index, and its applicability is analyzed by testing the influence of various factors.
(2) The signal damping factor is taken into account by the proposed WMPE algorithm, and thus the multiple phasors in the measured signal can be accurately estimated, even if the signal includes fundamental, multi-harmonic, and multi-interharmonic tones; covers the frequency range of 10–2000 Hz; and has non-zero damping factors.
(3) Several test scenarios are designed according to synchrophasor standards and literature studies on wideband phasor measurement. The test signals include wideband multi-components with different damping factors, noise, amplitudes or phase modulations, frequency deviations or ramps, and different interharmonic frequencies and transient changes. The test results confirm that the proposed method can accurately estimate wideband multi-component phasors, and has a short response time in these complex signal environments.

## 2. The Complete Process of the Proposed WMPE Algorithm

This section proposes the WMPE algorithm to realize phasor extraction from signals containing interharmonic tones. It includes three steps: signal component number identification, signal component frequency and damping factor estimation, and wideband multi-component phasor extraction. The first step proposes a robust index to identify the signal component number. Then, the methods in the last two steps take the signal damping factor into account. These two points are critical to improving the accuracy of estimating wideband multi-component phasors.

### 2.1. The Proposed Index for Identifying Signal Component Number

In this part, based on the singular value decomposition (SVD) of the Hankel matrix, an index is proposed to obtain the number of signal components from the noisy samples [44]. It starts with the dynamic voltage or current signal x(t) with the following model as

(1) $$x\left(t\right)=\sum_{i=1}^{M}s_{i}\left(t\right)+w\left(t\right)=\sum_{i=1}^{M}a_{i}\left(t\right)e^{\alpha_{i}t}\cos\left(2\pi f_{i}t+\phi_{i}\left(t\right)\right)+w\left(t\right),$$

where *M* is the number of the signal component $s_{i}\left(t\right)$, which denotes the fundamental, harmonic, or interharmonic tone; w(t) is the noise; and $a_{i}\left(t\right)$, $\alpha_{i}$, $f_{i}$, and $\phi_{i}\left(t\right)$ denote the amplitude, damping factor, frequency, and phase of the component $s_{i}\left(t\right)$. Two of these parameters form the phasor $p_{i}\left(t\right)=a_{i}\left(t\right)e^{j\phi_{i}\left(t\right)}$ to be estimated. Before doing so, the number of signal components must first be identified, which in this paper is obtained from the SVD of the Hankel matrix. When the signal x(t) is sampled with frequency $f_{s}$, the Hankel matrix is composed of $N=2N_{h}+1$ signal samples $x\left(-N_{h}T_{s}\right)\;\cdots ,\;x\left(N_{h}T_{s}\right)$ (the sampling interval $T_{s}=1/f_{s}$) in a data window $T_{w}$, i.e.,

(2) $$X=\left[\begin{array}{cccc} x(-N_{h}T_{s}) & \;x(\left(-N_{h}+1\right)T_{s}) & \cdots & \;x(\left(-N_{h}+L\right)T_{s})\\ x(\left(-N_{h}+1\right)T_{s}) & \;x(\left(-N_{h}+2\right)T_{s}) & \cdots & \;x(\left(-N_{h}+L+1\right)T_{s})\\ \vdots & \;\vdots & \ddots & \;\vdots \\ x(\left(N_{h}-L\right)T_{s}) & \;x(\left(N_{h}-L+1\right)T_{s}) & \cdots & \;x(N_{h}T_{s}) \end{array}\right],$$

where the parameter *L* is used to adjust the dimension of the matrix *X*, and is recommended to choose L∈[N/3,2N/3] to have a good suppression of noise in what follows [41].

Then, the matrix *X* is decomposed by the SVD [40] as

(3) $$X=U\Sigma V^{T},$$

where the left singular matrix $U\in R^{\left(N-L)\times (N-L\right)}$ and the right singular matrix $V\in R^{\left(L+1)\times (L+1\right)}$ are orthogonal matrices; their column vectors are $u_{i},\;v_{j}\;(1\leq i\leq \left(N-L\right),$1≤j≤(L+1)), respectively; the matrix $\Sigma \in R^{\left(N-L)\times (L+1\right)}$ is composed of singular values $\sigma_{i}$ in the diagonal and zero in other places (1≤i≤B,B=min{(N−L),(L+1)}, where min {a,b} denotes the smaller one between *a* and *b*); and $Y^{T}$ represents the transpose of the matrix *Y*. Based on the singular values arranged in a non-increasing form, the following index $G_{k}$ and the corresponding criterion are presented to obtain the signal component number *M*, i.e.,

(4) $$\begin{array}{cc} G_{k} & =\left(\sigma_{2k+3}+\sigma_{2k+4}\right)/\left(\sigma_{2k+1}+\sigma_{2k+2}\right),\;0\leq k\leq \left\lfloor B/2\right\rfloor -1,\\ \hat{M} & =k_{G\min}+1, \end{array}$$

where $k_{G\min}$ means the location of the minimum $G_{k}$, ⌊·⌋ denotes the round-down operation, and $\hat{Y}$ represents the estimate of *Y*. Note that each cosine or sine signal corresponds to two singular values, and the magnitude of the singular value is positively related to the component amplitude or noise intensity [45,46]. Then, the singular values of one signal component are close to those of another signal component, and the singular values of the noise are about the same amount; that is, the signal component produces larger singular values than the noise for having a larger amplitude. Therefore, the ratio index $G_{k}$ has a minimum value when the numerator is singular values for noise and the denominator is singular values for signal components. The proposed index and criterion are proved to be more robust than those of [44] in Appendix A, and require only 1/4 the computation amount of those in [44].

Equation (4) shows that the performance of the proposed index is closely related to the number and quantitative ratio of singular values of the Hankel matrix. In the following, the influence of various factors on the reliability and robustness of the proposed index and the criterion for determining the scope of application is discussed. The first type of factors includes different damping ratios, signal component amplitudes, and noise intensities, which can affect the quantitative relationship of singular values and thus the accuracy of the proposed criterion. Sampling frequency and time window length belong to another category and will affect the number of singular values and the signal components available for estimation. Then, five tests are designed to explore the influences of the above five factors, referring to [1,11,47] to set the test signal parameters in (5). The general settings include the nominal frequency $f_{0}=50$ Hz; the fundamental tone has amplitude $A_{1}=1$ p.u., frequency $f_{1}=f_{0}$, and phase $\phi_{1}$ randomly chosen in [−π,π]; the harmonics have amplitudes $A_{h}=0.1$ p.u., frequencies $f_{h}=hf_{0}$, phases $\phi_{h}\in \left[-\pi ,\pi \right]$, and maximum harmonic order $H=f_{s}/2/f_{0}-1$; the interharmonic tones have phases $\phi_{i}\in \left[-\pi ,\pi \right]$; and the dimension parameter $L=N_{h}$ has good noise immunity [41].

(5) $$\begin{array}{c} x\left(t\right)=\sum_{h=1}^{H}A_{h}e^{\alpha_{h}t}\cos\left(2\pi f_{h}t+\phi_{h}\right)+\sum_{i=1}^{M_{i}}A_{i}e^{\alpha_{i}t}\cos\left(2\pi f_{i}t+\phi_{i}\right)+w\left(t\right). \end{array}$$

Moreover, in each test, only one of the five factors changes, and the others remain unchanged as follows:

- T1: The damping ratios for all signal components, i.e., $\alpha_{h}$ and $\alpha_{i}$, increase from −1 to 1 with a step of 0.2; the interharmonic amplitudes $A_{i}=0.1$ p.u., frequencies $f_{i}=47+100\left(i-1\right)$, and number $M_{i}=50$; the signal-to-noise ratio (SNR) is 60 dB; the sampling frequency $f_{s}=10$ kHz; and the time window $T_{w}=3/f_{0}$. Then, this test signal contains a total of 149 signal components, i.e., the maximum component number ⌊B/2⌋ in (4), and the minimum frequency interval is only 3 Hz.
- T2: The interharmonic tones have amplitudes $A_{i}\in \left[0.02,\;0.2\right]$ p.u. with a step of 0.02 p.u.; frequencies $f_{i}=47+100\left(i-1\right)$, and number $M_{i}=50$; the damping ratios $\alpha_{h}=1$ and $\alpha_{i}=1$; the SNR is 60 dB; the sampling frequency $f_{s}=10$ kHz; and the time window $T_{w}=3/f_{0}$.
- T3: The SNR changes from 50 dB to 80 dB in a step of 5 dB; the damping ratios $\alpha_{h}=1$ and $\alpha_{i}=1$; the interharmonic tones have amplitudes $A_{i}=0.1$ p.u., frequencies $f_{i}=47+100\left(i-1\right)$, and number $M_{i}=50$; the sampling frequency $f_{s}=10$ kHz; and the time window $T_{w}=3/f_{0}$.
- T4: The sampling frequency $f_{s}$ increases from 5 kHz to 10 kHz with a step 1 kHz; the damping ratios $\alpha_{h}=1$ and $\alpha_{i}=1$; the interharmonic tones have amplitudes $A_{i}=0.1$ p.u., frequencies $f_{i}=47+100\left(i-1\right)$, and number $M_{i}=f_{s}/\left(4f_{0}\right)$; the SNR is 60 dB; and the time window $T_{w}=3/f_{0}$.
- T5: The time window length $c=T_{w}\cdot f_{0}$ changes from 2 to 7 in a step of 1; the damping ratios $\alpha_{h}=1$ and $\alpha_{i}=1$; the interharmonic tones have amplitudes $A_{i}=0.1$ p.u., frequencies $f_{i}=f_{b}+100\left(i-1\right),\;1\leq i\leq 50,\;f_{b}$ traverses {47}, {47, 70}, {20, 47, 70}, {20, 47, 70, 90}, and {10, 30, 47, 70, 90} for c=3,4,5,6,7, respectively, and number $M_{i}=50\left(c-2\right)$, i.e., the signal contains no interharmonic when *c* = 2; the SNR is 60 dB; and the sampling frequency $f_{s}=10$ kHz.

Note that each signal configuration is performed 1000 times, and the accuracy performance is defined as the proportion of $\hat{M}\geq \left(H+M_{i}\right)$, i.e., the estimated signal component number is not less than the set value to guarantee that the signal information can be fully extracted. The state-of-the-art maximum criterion (Max) in [45] and the commonly used threshold method (Thr) in [41] are also compared. For different damping ratios in T1, both the proposal and Max can have 100% accuracy to yield $\hat{M}\geq \left(H+M_{i}\right)$. The Thr method, on the other hand, always produces $\hat{M}<\left(H+M_{i}\right)$ and thus loses some signal information, as will be observed in the following tests. The results for tests T2–T5 are shown in Table 1, and three points can be observed: (1) The proposed index is more robust than the Max and Thr criteria; (2) When the amplitude ratios of interharmonics and harmonics belong to [0.8, 1.6], and the SNR is larger than 55 dB, the proposal has a good accuracy level, i.e., the proportion of $\hat{M}\geq \left(H+M_{i}\right)$ is over 99.9%; (3) The sampling frequency and time window length have little to do with the accuracy level, but are positively related to the number of signal components available to be estimated.

### 2.2. The Signal Component Frequency and Damping Factor Estimation Based on the Matrix Pencil

Given the estimated signal component number $\hat{M}$, a Hankel matrix with an improved SNR can be reconstructed by removing the singular values and vectors related to noise as

(6) $$\hat{X}=U_{s}\Sigma_{s}V_{s}^{T},$$

where $\hat{X}\in R^{\left(N-L)\times (L+1\right)}$ and $U_{s}\in R^{\left(N-L\right)\times 2\hat{M}},\;V_{s}\in R^{\left(L+1\right)\times 2\hat{M}}$ are composed of the first $2\hat{M}$ column vectors of *U* and *V*, respectively; and the diagonal matrix $\Sigma_{s}\in R^{2\hat{M}\times 2\hat{M}}$ contains the singular values from signal components in Σ.

In order to estimate the signal component frequency and damping factor using the matrix pencil, the signal in (1) is discreted and then rewritten with phasor $p_{i}\left(t\right)=a_{i}\left(t\right)e^{j\phi_{i}\left(t\right)}$ as

(7) $$\begin{array}{c} x\left(nT_{s}\right)=\sum_{i=1}^{M}\left(0.5p_{i}\left(nT_{s}\right)z_{i}^{n}+0.5p_{i}^{*}\left(nT_{s}\right){\left(z_{i}^{*}\right)}^{n}\right)+w\left(nT_{s}\right), \end{array}$$

where $Y^{*}$ denotes the conjugate of complex number *Y*, and $z_{i}=e^{\left(\alpha_{i}+j2\pi f_{i}\right)T_{s}}$. With (7), the Hankel matrix in (6) can be decomposed as

(8) $$\begin{array}{cc} \hat{X} & =\left[\begin{array}{cccccc} z_{1}^{0} & \cdots & z_{\hat{M}}^{0} & {\left(z_{1}^{*}\right)}^{0} & \cdots & {\left(z_{\hat{M}}^{*}\right)}^{0}\\ z_{1} & \cdots & z_{\hat{M}} & z_{1}^{*} & \cdots & z_{\hat{M}}^{*}\\ \vdots & \ddots & \vdots & \vdots & \ddots & \vdots \\ z_{1}^{\left(2N_{h}-L\right)} & \cdots & z_{\hat{M}}^{\left(2N_{h}-L\right)} & {\left(z_{1}^{*}\right)}^{\left(2N_{h}-L\right)} & \cdots & {\left(z_{\hat{M}}^{*}\right)}^{\left(2N_{h}-L\right)} \end{array}\right]\\ \; & \times \left[\begin{array}{cccccc} 0.5p_{1} & \; & & \; & & \\ \; & \ddots & \; & & \; & \\ \; & \; & 0.5p_{\hat{M}} & \; & & \\ \; & \; & & 0.5p_{1}^{*} & \; & \\ \; & \; & & \; & \ddots & \\ \; & \; & & \; & & 0.5p_{\hat{M}}^{*} \end{array}\right]\\ \; & \times \left[\begin{array}{cccc} z_{1}^{\left(-N_{h}\right)} & z_{1}^{\left(-N_{h}+1\right)} & \cdots & z_{1}^{\left(-N_{h}+L\right)}\\ \vdots & \vdots & \ddots & \vdots \\ z_{\hat{M}}^{\left(-N_{h}\right)} & z_{\hat{M}}^{\left(-N_{h}+1\right)} & \cdots & z_{\hat{M}}^{\left(-N_{h}+L\right)}\\ {\left(z_{1}^{*}\right)}^{\left(-N_{h}\right)} & {\left(z_{1}^{*}\right)}^{\left(-N_{h}+1\right)} & \cdots & {\left(z_{1}^{*}\right)}^{\left(-N_{h}+L\right)}\\ \vdots & \vdots & \ddots & \vdots \\ {\left(z_{\hat{M}}^{*}\right)}^{\left(-N_{h}\right)} & {\left(z_{\hat{M}}^{*}\right)}^{\left(-N_{h}+1\right)} & \cdots & {\left(z_{\hat{M}}^{*}\right)}^{\left(-N_{h}+L\right)} \end{array}\right]=Z_{L}PZ_{R}. \end{array}$$

Then, two matrices $\hat{X_{1}},\;\hat{X_{2}}\in R^{\left(N-L-1)\times (L+1\right)}$ that are obtained by, respectively, removing the last and the first column vector of $\hat{X}$ can be decomposed as

(9) $$\begin{array}{cc} \hat{X_{1}} & =Z_{L}PZ_{R1},\\ \hat{X_{2}} & =Z_{L}PZ_{R2}, \end{array}$$

where $Z_{R1},\;Z_{R2}\in C^{2\hat{M}\times L}$ are formed by removing the last column vector and the first column vector of $Z_{R}$, respectively, and there is

(10) $$Z_{R2}=ZZ_{R1},$$

where the diagonal matrix $Z\in C^{2\hat{M}\times 2\hat{M}}$ is composed of $z_{i}\;\left(1\leq i\leq 2\hat{M}\right)$, and explains that the element $\hat{X_{2}}\left(i,j\right)$ is one sampling interval ahead of the element $\hat{X_{1}}\left(i,j\right)$. Then, the matrix pencil form $\hat{X_{2}}-\lambda \left(I\right)\hat{X_{1}}=Z_{L}P\left(Z-\lambda \left(I\right)\right)Z_{R1}$ takes the matrix *Z* as a solution [48], and a simplified form can be deduced as

(11) $$\hat{Z}=\mathrm{eig}\left(\hat{X_{1}^{+}}\hat{X_{2}}\right)=\mathrm{eig}\left({\left(U_{s}\Sigma_{s}V_{s1}^{T}\right)}^{+}U_{s}\Sigma_{s}V_{s2}^{T}\right)=\mathrm{eig}\left({\hat{V}}_{s1}^{+}{\hat{V}}_{s2}\right),$$

where eig(Y) denotes obtaining the eigenvalues of the matrix *Y*, $Y^{+}$ represents the Moore–Penrose pseudoinverse of the matrix *Y*, and $V_{s1},\;V_{s2}\in R^{L\times 2\hat{M}}$ are formed by removing the last row vector and the first-row vector of $V_{s}$, respectively. Then, the frequency and damping factor of each cosine or sine signal component are obtained by

(12) fi^=Im(loge(z^i))2πTs,αi^=Re(loge(z^i))Ts,1≤i≤M^,

where Im(Y) and Re(Y) represent the imaginary and real parts of the complex number *Y*; $\log_{e}\left(\cdot \right)$ denotes the natural logarithmic function.

### 2.3. The Wideband Multi-Component Phasor Estimation Based on the Modified Least-Squares Algorithm

Combining the decomposition of $\hat{X}$ in (8) and the estimated frequency and damping factor in (12), the phasor matrix *P* can be obtained by the modified least-squares algorithm as

(13) $$\hat{P}=\mathrm{eig}\left({\left(Z_{L}^{H}Z_{L}\right)}^{-1}Z_{L}^{H}\hat{X}Z_{R}^{H}{\left(Z_{R}Z_{R}^{H}\right)}^{-1}\right),$$

where $Y^{H},\;Y^{-1}$ denote the Hermitian and inverse operations of the matrix *Y*. Finally, the amplitude and phase for each cosine or sine signal component can be calculated from the phasor estimation $\hat{P}$, i.e.,

(14) $$\begin{array}{cc} {\hat{a}}_{i}= & 2|{\hat{P}}_{i,i}|,\\ {\hat{\phi }}_{i}= & ∠{\hat{P}}_{i,i},\;1\leq i\leq \hat{M}, \end{array}$$

where ${\hat{P}}_{i,i}$ denotes the *i*-th diagonal element of the phasor matrix $\hat{P}$.

**Figure 1:**
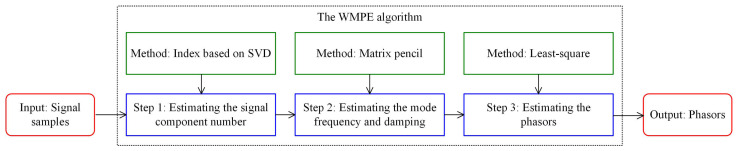
The block diagram of the proposed WMPE algorithm.

As seen in Figure 1, in conclusion, the proposed WMPE algorithm includes three crucial steps to extract all the phasors in the measured signal. The first step, the accurate identification of the frequency number by the proposed index and criterion, is very important for the subsequent estimation of the frequency, damping factor, amplitude, and phase. Therefore, its effectiveness and scope of application are specifically analyzed in Section 2.1. In the following section, numerical and experimental tests are carried out to verify the phasor estimation accuracy of the proposed WMPE algorithm.

## 3. Numerical Tests

This section designs seven scenarios referring to [1,11,47] to test the accuracy and transient response performance of the proposed WMPE algorithm under the condition of different damping factors, amplitudes, phases, and frequencies of the signal components. Three state-of-the-art algorithms, HI−MP [41], MEMO−ESPRIT [49], and SD−ESPRIT [50], are employed for comparison. The definition of total vector error (TVE), response time, and reporting rate $f_{\mathrm{re}}$ refers to the IEC/IEEE standard [11]. Some general parameter settings include the sampling frequency $f_{s}=10\;$ kHz, the reporting rate $f_{\mathrm{re}}=$50 frames/s, the nominal frequency $f_{0}=50$ Hz, and the data window $T_{w}=3/f_{0}$ to achieve signal component number identification under the noise interference, and the dimension parameter $L=N_{h}$ to yield good noise immunity [41].

Case A: Various damping factors

The first case is carried out to test the accuracy performance of the proposed WMPE and the compared MEMO−ESPRIT and HI−MP algorithms under various damping factors. The test signal has the form of (5). The fundamental amplitude, frequency, and phase are $A_{1}=1\;p.u.,\;f_{1}=f_{0},\;\phi_{1}\in \left[-\pi ,\pi \right]$, respectively; the harmonic amplitude, frequency, and phase are $A_{h}=0.1\;p.u.,\;f_{h}=hf_{0},\;\phi_{h}\in \left[-\pi ,\pi \right]\;\left(h\in \left[2,\;H\right]\right)$, respectively; the maximum harmonic order H=13 [32]; the interharmonic amplitude, frequency, and phase are $A_{i}=0.1\;p.u.,\;f_{i}=35+100\left(i-1\right),\;\phi_{i}\in \left[-\pi ,\pi \right]$, respectively; and the interharmonic number $M_{i}=20$. Then, the signal contains a total of 33 components for each test, and the maximum component frequency is close to 2000 Hz. In this scenario, all the damping factors $\alpha_{h}$ and $\alpha_{i}$ increase from −1 to 1 in a step of 0.1 in every test. The added white noise w(t) has an SNR of 60 dB. The settings of the noise intensity of 60 dB and the maximum harmonic order H=13 [32] remain the same across all the seven cases and will not be repeatedly stated.

**Figure 2:**
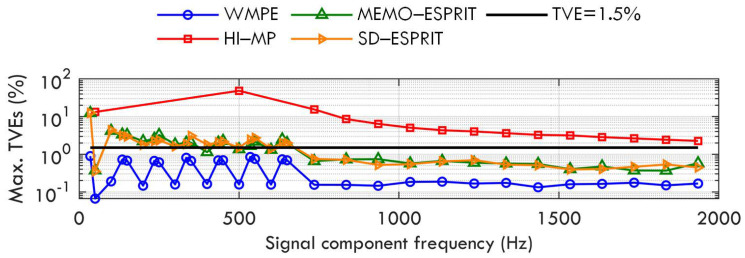
Maximum TVEs obtained by WMPE, HI−MP, MEMO−ESPRIT, and SD−ESPRIT algorithms under various damping factors.

Figure 2 shows the maximum TVEs of the proposed WMPE, the HI−MP, MEMO−ESPRIT, and SD−ESPRIT algorithms, as well as a TVE reference limit of 1.5% according to [11]. The TVE limit will also be plotted in Cases B to F to check the accuracy of the four algorithms. As seen in Figure 2, the proposed WMPE algorithm performs much better accuracy than the three compared algorithms for all signal components. When the damping factors of all signal components change between [−1,1], the maximum TVEs of the proposed WMPE are always less than 1.5%, which indicates that the proposal has good robustness to damping factor changes. In addition, the red curve shows that the HI−MP algorithm cannot estimate the signal components with a frequency interval below 50 Hz when the time window is only three nominal cycles, which can also be observed in the following tests. The blue curve indicates that the proposed WMPE algorithm produces fewer TVEs than the MEMO−ESPRIT and SD−ESPRIT algorithms because it considers the influence of the damping factor. Note that there are 33 tones in the test signal, and the frequency interval for adjacent components below 650 Hz is less than 50 Hz, while that for tones above 650 Hz is 100 Hz. Therefore, there will be some error peaks for phasors close in frequency when using the proposed WMPE algorithm to estimate phasors.

Case B: Amplitude modulation of fundamental and harmonics

Cases B and C aim to test the four algorithms under dynamic conditions. When there is amplitude modulation for fundamental and harmonics accompanied by the increasing amplitude for interharmonics, the test signal is given as

(15) $$\begin{array}{c} x\left(t\right)=\sum_{h=1}^{H}A_{h}\left(1+0.1\cos\left(2\pi f_{m}t\right)\right)\cos\left(2\pi f_{h}t+\phi_{h}\right)+\sum_{i=1}^{M_{i}}A_{i}e^{\alpha_{i}t}\cos\left(2\pi f_{i}t+\phi_{i}\right)+w\left(t\right), \end{array}$$

where the fundamental has amplitude, frequency, and phase are $A_{1}=1\;p.u.,\;f_{1}=f_{0},\;$$\phi_{1}\in \left[-\pi ,\pi \right]$; the harmonics have amplitude, frequency, and phase of $A_{h}=0.1\;p.u.,$$\;f_{h}=hf_{0},$$\;\phi_{h}\in \left[-\pi ,\pi \right]\;\left(h\in \left[2,\;H\right]\right)$; all the amplitudes of the fundamental and harmonics are modulated at frequency $f_{m}$, which changes from 0.1 Hz to 2 Hz by 0.1 Hz in each implementation; the interharmonic amplitude, damping factor, frequency, and phase are $A_{i}=0.1\;p.u.,\;\alpha_{i}=1,\;f_{i}=35+100\left(i-1\right),\;\phi_{i}\in \left[-\pi ,\pi \right]$, respectively; and the interharmonic number $M_{i}=20$.

**Figure 3:**
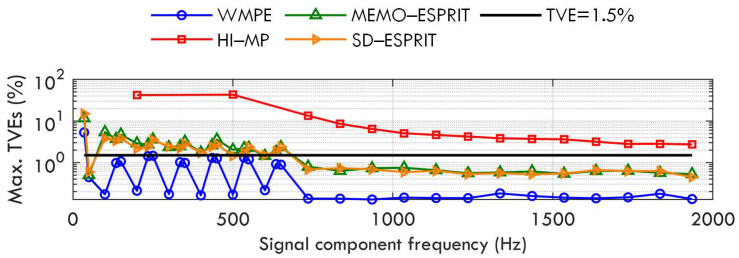
Maximum TVEs obtained by WMPE, HI−MP, MEMO−ESPRIT, and SD−ESPRIT algorithms under amplitude modulation of fundamental and harmonics.

The amplitude modulation frequency $f_{m}$ changes in the range of [0.1,2] Hz. The maximum estimation error of the WMPE, HI−MP, MEMO−ESPRIT, and SD−ESPRIT algorithms is shown in Figure 3. It is observed that the proposal still yields a better estimation than the other three algorithms under this dynamic condition. However, Figure 3 also shows that for the proposed WMPE algorithm, the maximum TVE of the first component, i.e., a sub-synchronous component of 35 Hz, is more than 1.5%. This is because the dynamic fundamental produces more spectral leakage and a worse effect on the estimation of the sub-synchronous phasor than the steady fundamental (similar conclusions can be observed when estimating the super-synchronous phasor under the interference of the dynamic fundamental).

Case C: Phase modulation of fundamental and harmonics

In this test, the signal contains fundamental and harmonics having phase modulation and interharmonics with positive damping factor, i.e.,

(16) $$\begin{array}{c} x\left(t\right)=\sum_{h=1}^{H}A_{h}\cos\left(2\pi f_{h}t+\phi_{h}+0.1\cos\left(2\pi f_{m}t-\pi \right)\right)+\sum_{i=1}^{M_{i}}A_{i}e^{\alpha_{i}t}\cos\left(2\pi f_{i}t+\phi_{i}\right)+w\left(t\right), \end{array}$$

where $A_{1}=1\;p.u.,\;f_{1}=f_{0},\;\phi_{1}\in \left[-\pi ,\pi \right]$, and $A_{h}=0.1\;p.u.,\;f_{h}=hf_{0},\;\phi_{h}\in \left[-\pi ,\pi \right]\;$(h∈[2,H]); all the phases of the fundamental and harmonics are added with a modulation part at frequency $f_{m}$, which increases from 0.1 Hz to 2 Hz by 0.1 Hz in each test. For interharmonic tones, there are $A_{i}=0.1\;p.u.,\;\alpha_{i}=1,\;f_{i}=35+100\left(i-1\right),\;\phi_{i}\in \left[-\pi ,\pi \right],\;M_{i}=20$.

**Figure 4:**
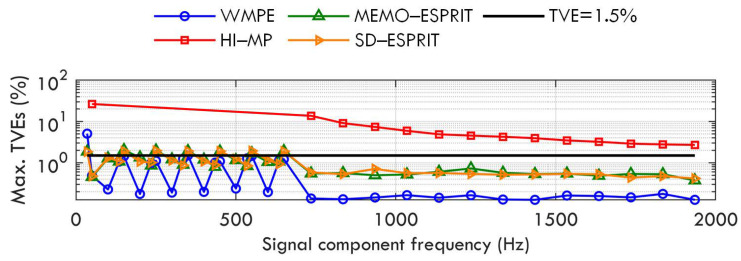
Maximum TVEs obtained by WMPE, HI−MP, MEMO−ESPRIT, and SD−ESPRIT algorithms under phase modulation of fundamental and harmonics.

As seen in Figure 4, the test results show that even when the phase modulation frequency $f_{m}$ is up 2 Hz, the WMPE, MEMO−ESPRIT, and SD−ESPRIT algorithms can have good phasor estimation of all the signal components, whereas the proposal achieves a better estimation than the MEMO−ESPRIT and SD−ESPRIT algorithms, and produces a TVE below 1.5% for most components. However, for the sub-synchronous phasor (and the super-synchronous phasor), the proposal achieves an estimation accuracy similar to that in Case B, because it is affected by fundamental phase modulation.

Case D: Frequency deviation of fundamental and harmonics

In this test, the frequency deviation of fundamental and harmonics and the increasing amplitude of the interharmonic tones are considered. Then, the test signal has the form of

(17) $$x\left(t\right)=\sum_{h=1}^{H}A_{h}\cos\left(2\pi hf_{d}t+\phi_{h}\right)+\sum_{i=1}^{M_{i}}A_{i}e^{\alpha_{i}t}\cos\left(2\pi f_{i}t+\phi_{i}\right)+w\left(t\right),$$

where the amplitude and phase of the fundamental and harmonics (h∈[2,H]) are $A_{1}=1\;p.u.,\;A_{h}=0.1\;p.u.,\;\phi_{1},\phi_{h}\in \left[-\pi ,\pi \right]$; the fundamental frequency $f_{d}$ changes from 49.5 Hz to 50.5 Hz [32] in a step of 0.1 Hz, while the harmonic frequency keeps an integer multiple of $f_{d}$; and the amplitude, damping factor, frequency, phase, and number of the interharmonic tones are $A_{i}=0.1\;p.u.,\;\alpha_{i}=1,\;f_{i}=30+100\left(i-1\right),\;\phi_{i}\in \left[-\pi ,\pi \right],\;M_{i}=20$.

**Figure 5:**
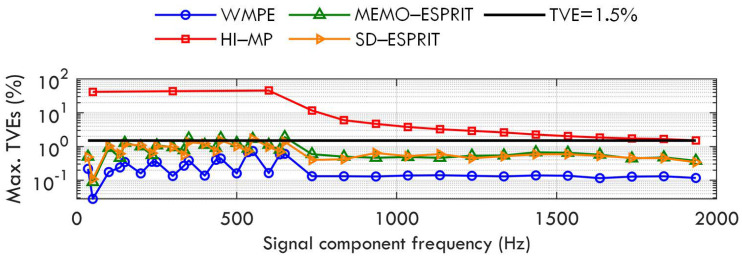
Maximum TVEs obtained by WMPE, HI−MP, MEMO−ESPRIT, and SD−ESPRIT algorithms under frequency deviation of fundamental and harmonics.

As Figure 5 shows, the proposal performs better than the HI−MP, MEMO−ESPRIT, and SD−ESPRIT algorithms under the deviated fundamental and harmonic frequencies. Figure 5 also indicates that the estimation errors of odd harmonics increase with the harmonic order. This is because the deviated harmonics become closer to the adjacent interharmonic tones, which deteriorates their mutual spectral leakage interference.

Case E: Frequency ramp change of fundamental and harmonics

The dynamic frequency change may cause a larger estimation error than the steady frequency deviation when covering the same frequency range. Therefore, this test signal considers the dynamic change in the fundamental and harmonic frequencies, i.e.,

(18) $$\begin{array}{c} x\left(t\right)=\sum_{h=1}^{H}A_{h}\cos\left(2\pi hf_{d}t+\phi_{h}+\pi R_{h}t^{2}\right)+\sum_{i=1}^{M_{i}}A_{i}e^{\alpha_{i}t}\cos\left(2\pi f_{i}t+\phi_{i}\right)+w\left(t\right), \end{array}$$

where $A_{1}=1\;p.u.,\;f_{d}=49.5\;\mathrm{Hz},\;\phi_{1}\in \left[-\pi ,\pi \right]$; $A_{h}=0.1\;p.u.,\;hf_{d}=49.5h\;\mathrm{Hz}$, $\;\phi_{h}\in \left[-\pi ,\pi \right]\;\left(h\in \left[2,\;H\right]\right)$; the fundamental frequency increases from 49.5 Hz to 50.5 Hz in a second, i.e., $R_{1}=1\;$ Hz/s, while the harmonic frequencies change from 49.5*h* Hz to 50.5*h* Hz, i.e., $R_{h}=h\;$ Hz/s; and the interharmonic amplitude, damping factor, frequency, phase, and number are $A_{i}=0.1\;p.u.,\;\alpha_{i}=1,\;f_{i}=30+100\left(i-1\right),\;\phi_{i}\in \left[-\pi ,\pi \right],\;M_{i}=20$, respectively.

**Figure 6:**
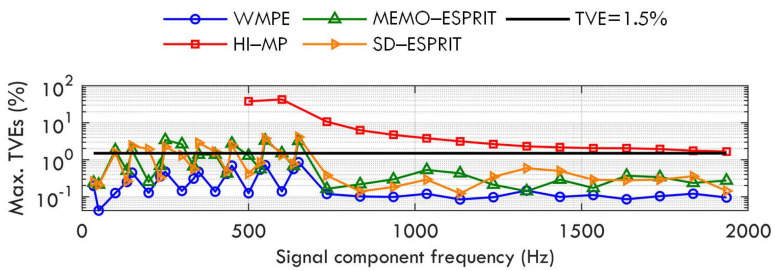
Maximum TVEs obtained by WMPE, HI−MP, MEMO−ESPRIT, and SD−ESPRIT algorithms under frequency ramp change of fundamental and harmonics.

As shown in Figure 6, the proposal realizes similar phasor estimation accuracy as in Case D for all signal components, while MEMO−ESPRIT and SD−ESPRIT may produce larger error in this case than in Case D. This again confirms that the proposed WMPE algorithm has good dynamic performance.

Case F: Different interharmonic frequency

For Cases A to E, the first interharmonic component is always sub-synchronous oscillation. In this case, the super-synchronous oscillation is also employed to test the four algorithms. The test signal is given as

(19) $$x\left(t\right)=\sum_{h=1}^{H}A_{h}\cos\left(2\pi f_{h}t+\phi_{h}\right)+\sum_{i=1}^{M_{i}}A_{i}e^{\alpha_{i}t}\cos\left(2\pi f_{i}t+\phi_{i}\right)+w\left(t\right),$$

where the fundamental amplitude, frequency, and phase are $A_{1}=1\;p.u.,\;f_{1}=f_{0},$$\;\phi_{1}\in \left[-\pi ,\pi \right]$, respectively; the harmonic amplitude, frequency, and phase are $A_{h}=0.1\;p.u.,$$\;f_{h}=hf_{0},\;\phi_{h}\in \left[-\pi ,\pi \right]\;\left(h\in \left[2,\;H\right]\right)$, respectively; the interharmonic amplitude, damping factor, frequency, phase, and number are $A_{i}=0.1\;p.u.,$$\;\alpha_{i}=1,\;f_{i}=f_{b}+100\left(i-1\right),\;\phi_{i}\in \left[-\pi ,\pi \right],\;M_{i}=20$, respectively; and the first interharmonic frequency $f_{b}\in \left\{10,\;15,\;20,\;25,\;30,\;35,\;65,\;70,\;75,\;80,\;85,\;90\right\}$.

**Figure 7:**
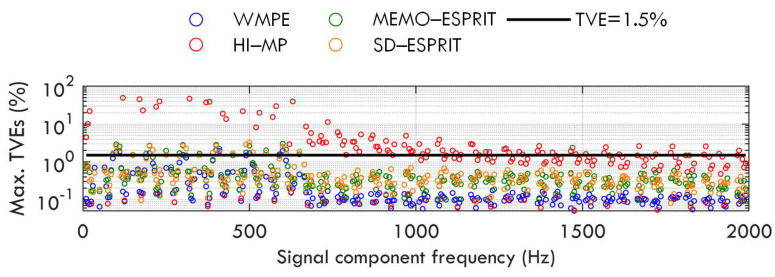
Maximum TVEs obtained by WMPE, HI−MP, MEMO−ESPRIT, and SD−ESPRIT algorithms under different interharmonic frequencies.

In each test, the signal contains 33 components, and one value from the set of $f_{b}$ is chosen to determine the interharmonic frequencies. All set values of $f_{b}$ are traversed. The maximum TVEs obtained by the proposed WMPE, HI−MP, MEMO−ESPRIT, and SD−ESPRIT algorithms are shown in Figure 7. For the WMPE, MEMO−ESPRIT, and SD−ESPRIT algorithms, the accuracy of estimating sub-synchronous and super-synchronous phasors is about the same. However, when the frequency interval of two signal components is as small as 10 Hz, e.g., 200 Hz and 210 Hz, their maximum estimation errors may be more than 1.5%, as seen in Figure 7.

Case G: Transient response

This case is carried out to test the transient response time of the four algorithms, and hence the signal contains a transient change, i.e.,

(20) $$\begin{array}{c} x\left(t\right)=\left\{\begin{array}{cc} \; & \sum_{h=1}^{H}A_{h}\cos\left(2\pi f_{h}t+\phi_{h}\right)+w\left(t\right),\;t<t_{0},\\ \; & \sum_{h=1}^{H}A_{h}\cos\left(2\pi f_{h}t+\phi_{h}\right)\\ \; & +\sum_{i=1}^{M_{i}}A_{i}e^{\alpha_{i}t}\cos\left(2\pi f_{i}t+\phi_{i}\right)+w\left(t\right),\;t\geq t_{0}, \end{array}\right. \end{array}$$

where the fundamental has amplitude, frequency, and phase are $A_{1}=1\;p.u.,\;f_{1}$$=f_{0},$$\;\phi_{1}\in \left[-\pi ,\pi \right]$; the harmonic has amplitude, frequency, and phase are $A_{h}=0.1\;p.u.,$$\;f_{h}=hf_{0},$$\;\phi_{h}\in \left[-\pi ,\pi \right]\;\left(h\in \left[2,\;H\right]\right)$; and the interharmonic has an amplitude, damping factor, frequency, phase, and numbers of $A_{i}=0.1\;p.u.,\;\alpha_{i}=1,\;f_{i}=35+100\left(i-1\right),\;\phi_{i}\in \left[-\pi ,\pi \right],\;M_{i}=20$.

As seen in (20), at the time $t_{0}=0$ in this paper, there are $M_{i}$ interharmonic tones with increasing amplitude added to the signal x(t). Table 2 shows the response times of the proposed WMPE algorithm, defined as the duration in which the estimation error exceeds 1% [11] when a transient occurs. For the HI−MP, MEMO−ESPRIT, and SD−ESPRIT algorithms, the response times are not available for some components because the estimation errors are also greater than 1% for signals without a transient event. However, the proposed WMPE achieves the response times below the data window length $T_{w}=60$ ms for all signal components.

## 4. Experimental Test

**Figure 8:**
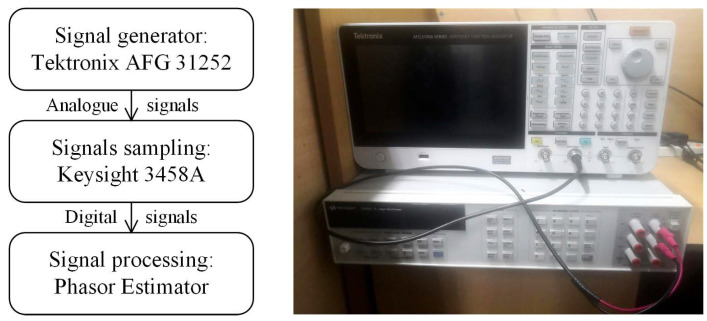
The block diagram (**the left**) and the photo (**the right**) of the constructed physical platform.

In this section, an experimental test is performed to verify the performance of the proposed WMPE algorithm under the physical environment. As shown in Figure 8, the experimental platform mainly includes the devices that generate, sample, and process the test signals; that is, the test signals are physically generated by the Tektronix AFG 31252, sampled by the Keysight 3458A, and processed on a computer by the proposed WMPE and the compared HI−MP, MEMO−ESPRIT, and SD−ESPRIT algorithms. The results are wideband multiplier phasors extracted by these algorithms from the signal samples. The Tektronix AFG 31252, on the other hand, is used because it can output signals that are clear to the real power grid signals according to the formulas entered.In this section, an experimental platform is constructed with an arbitrary waveform signal generator (Tektronix AFG 31252) and a digital voltmeter (Keysight 3458A) to test the proposed WMPE algorithm. As shown in Figure 8, the Tektronix AFG 31252 is employed to produce signals according to the entered formulas, and the Keysight 3458A is used to sample signals. Finally, the signal samples are processed using the WMPE, HI−MP, MEMO−ESPRIT, and SD−ESPRIT algorithms. The result is wideband multiplier phasors.

**Figure 9:**
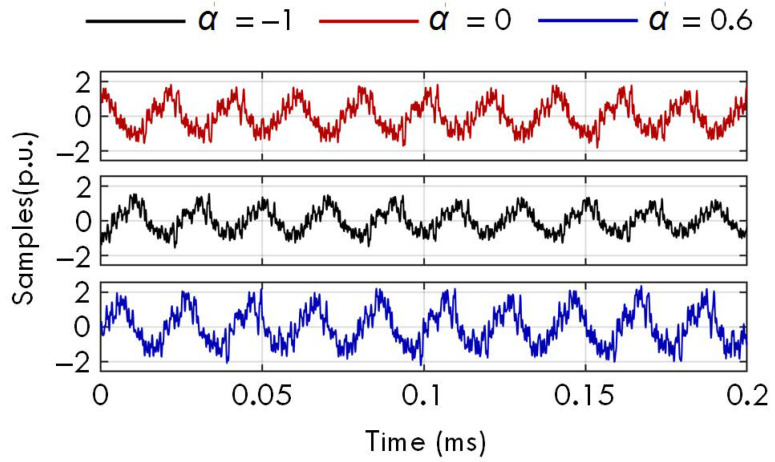
The samples of 3 test signals in the experimental test.

The signal form and settings in Case A are also used in this test. Namely, each test signal contains the fundamental, 12 harmonics, and 20 interharmonics, and the damping factors of these 33 components increase from −1 to 1 in steps of 0.2. Therefore, there are 11 test signals, and Figure 9 shows 3 of them. Then, these samples of all 11 distorted signals are used as the input data of the proposed WMPE, HI−MP, MEMO−ESPRIT and SD−ESPRIT algorithms to estimate the amplitudes and phases of all 33 signal components. To describe the estimation accuracy, the signal residual index in a time window is employed, i.e.,

(21) $$\begin{array}{c} \mathrm{Res}=\sqrt{\sum_{n=-N_{h}}^{N_{h}}{\left(x\left(nT_{s}\right)-\sum_{i=1}^{\hat{M}}{\hat{s}}_{i}\left(nT_{s}\right)\right)}^{2}/\sum_{n=-N_{h}}^{N_{h}}x^{2}\left(nT_{s}\right)}, \end{array}$$

where $x(nT_{s})$ denotes the signal samples, and ${\hat{s}}_{i}\left(nT_{s}\right)$ presents the constructed samples with the estimated components’ parameters obtained by different estimators.

**Figure 10:**
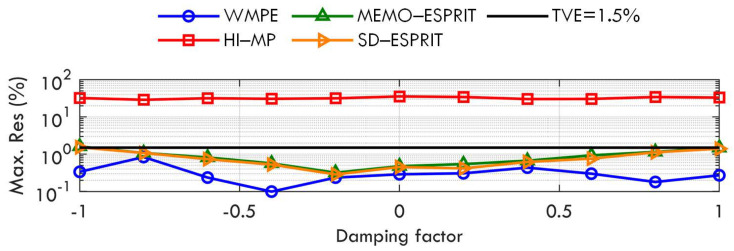
Maximum Res obtained by WMPE, HI−MP, MEMO−ESPRIT, and SD−ESPRIT algorithms under experimental test.

Figure 10 shows the variation of the maximum residual with damping factor obtained by the four algorithms. It is observed that the proposal yields maximum Res below 1.5%, and still achieves the best estimation for all damping factors in the experimental test, proving the effectiveness of the proposed WMPE algorithm.

## 5. Discussion

The numerical and experimental results show that the proposed WMPE algorithm yields a maximum total vector error of less than 1.5%, achieving a more accurate phasor estimation than existing methods under various signal environments. The proposed index and criterion can well identify the frequency number from the signal samples. Then, the modified matrix pencil and least-squares algorithm can accurately estimate the phasors in a wide frequency range and complicated signal environment. The obtained phasor data can be used to detect the oscillation source, improve the power quality by filtering the harmonic and interharmonic tones, help build accurate models of power electronics for better control performance, etc. However, the accuracy of the proposed WMPE algorithm is not guaranteed when the energy difference between the broadband signal components is large or when the noise is intense.

## 6. Conclusions

With the proliferation of power electronic devices and renewable energy generation, the signal condition of the power system becomes more complicated as they introduce harmonic and interharmonic tones into the power system over a wide frequency range and with non-zero damping. Then, considering the above signal characteristics, a wideband multi-component phasor estimator is proposed in this paper. Under the designed steady and dynamic signal environments, the proposal is able to achieve a maximum estimation error below 1.5%. It is more accurate than the existing algorithms. In particular, because the proposed method is more accurate and robust for identifying all the signal components, it provides a lower estimation error than the compared HI−MP algorithm. On the other hand, by considering the influence of the signal damping factor, the proposed method achieves more accurate wideband multi-component phasor estimation than the two ESPRIT algorithms. Moreover, the experimental results show that the proposed WMPE algorithm produces the smallest residual energy among the four algorithms, verifying it is of great application potential. However, it is worth noting that further accuracy improvement is needed for estimating the sub-/super-synchronous phasors under dynamic fundamental conditions and for estimating adjacent phasors that are close in frequency.

## Figures and Tables

**Table 1:** The probability that the proposed index and the compared Max and Thr methods can identify the number of all signal components, i.e., the proportion of $\hat{M}\geq \left(H+M_{i}\right)$ (%).

**T2** **(**$A_{i}$**)**	**0.02**	**0.04**	**0.06**	**0.08–0.14**	**0.16**	**0.18**	**0.2**
**Min**	0.3	14.3	84.7	100.0	99.9	98.6	97.2
**Max**	0.3	11.7	78.9	100.0	99.8	97.1	94.9
**Thr**	0	0	0	0	0	0	0
**T3 (SNR)**	**50**	**55**	**60**	**65**	**70**	**75**	**80**
**Min**	80.2	99.9	100.0	100.0	100.0	100.0	100.0
**Max**	72.8	99.4	100.0	100.0	100.0	100.0	100.0
**Thr**	0	0	0	0	0	0	0
**T4 ($f_{s}$)**	**5 k**	**6 k**	**7 k**	**8 k**	**9 k**	**10 k**	
**Min**	99.6	99.8	100.0	99.9	100.0	100.0	
**Max**	99.5	99.8	100.0	99.9	100.0	100.0	
**Thr**	0	0	0	0	0	0	
**T5 (*c*)**	**2**	**3**	**4**	**5**	**6**	**7**	
**Min**	100.0	96.1	99.9	100.0	100.0	100.0	
**Max**	100.0	94.0	99.3	100.0	100.0	100.0	
**Thr**	0	0	0	0	0	0	

**Table 2:** The response time (ms) obtained by the WMPE algorithm.

Order *h*	1	2	3	4	5	6	7
WMPE	58.5	58.6	58.7	57.9	58.7	57.9	58.7
Order *h*	8	9	10	11	12	13	
WMPE	57.9	58.7	57.9	58.7	57.9	58.7	

## Data Availability

The data that support the findings of this study are available from the corresponding author upon reasonable request.

## Abbreviations

The following abbreviations are used in this manuscript:

PMU	Phasor measurement unit
FFT	Fast Fourier transform
DFT	Discrete Fourier transform
LS	Least squares
WMPE	Wideband multi-component phasor estimator
SVD	Singular value decomposition
SNR	Signal–noise ratio
Max	Maximum criterion
Thr	Threshold method
MEMO	Modified exact model order
ESPRIT	Estimation of signal parameters using rotational invariance technique
HI−MP	Harmonic and interharmonic phasor estimation using matrix pencil

## Appendix A

This section proves the proposed index and criterion are more robust than those in [44]. The following equations give the index $R_{k}$ and criterion in [44].

(A1) $$\begin{array}{cc} R_{k} & =\sigma_{k+2}/\sigma_{k},\;0\leq k\leq B-2,\\ \hat{M} & =\lceil k_{R\min}/2\rceil , \end{array}$$

where $\sigma_{k}$ is the *k*-th diagonal element of matrix Σ in (3), $k_{R\min}$ denotes the location of minimum $R_{k}$, and ⌈·⌉ represents the round-up operation. The proposed index $G_{k}$ and criterion in (4) are more robust than that in [44] when the difference between the signal components and noise of $G_{k}$ is larger than that of $R_{k}$, i.e., $\Delta_{G}=G_{M-2}-G_{M-1}$ is larger than $\Delta_{R}=$ min $\{R_{2M-3},\;R_{2M-2}\}-$ max $\left\{R_{2M-1},\;R_{2M}\right\}$ where min {a,b} and max {a,b}, respectively, mean the smaller and bigger ones of *a* and *b*. Then, it is necessary to prove that $\Delta =\Delta_{G}-\Delta_{R}\geq 0$ where

(A2) $$\Delta =\left\{\begin{array}{cc} \Delta_{G}-\left(R_{2M-2}-R_{2M-1}\right), & R_{2M-2}\leq R_{2M-3},R_{2M}\leq R_{2M-1},\\ \Delta_{G}-\left(R_{2M-3}-R_{2M-1}\right), & R_{2M-3}<R_{2M-2},R_{2M}\leq R_{2M-1},\\ \Delta_{G}-\left(R_{2M-2}-R_{2M}\right), & R_{2M-2}\leq R_{2M-3},R_{2M-1}<R_{2M},\\ \Delta_{G}-\left(R_{2M-3}-R_{2M}\right), & R_{2M-3}<R_{2M-2},R_{2M-1}<R_{2M}. \end{array}\right.$$

The first equation in (A2) is taken as an example to show that there is always Δ≥0. As $R_{2M-2}\leq R_{2M-3}$ results in $\sigma_{2M-1}/\sigma_{2M}\geq \sigma_{2M-3}/\sigma_{2M-2}$, and $R_{2M}\leq R_{2M-1}$ results in $\sigma_{2M+2}/\sigma_{2M+1}\leq \sigma_{2M}/\sigma_{2M-1}$, there is

(A3) $$\begin{array}{cc} \Delta & =G_{M-2}-G_{M-1}-\left(R_{2M-2}-R_{2M-1}\right)\\ \; & =\frac{\sigma_{2M-1}+\sigma_{2M}}{\sigma_{2M-3}+\sigma_{2M-2}}-\frac{\sigma_{2M+1}+\sigma_{2M+2}}{\sigma_{2M-1}+\sigma_{2M}}-\left(\frac{\sigma_{2M}}{\sigma_{2M-2}}-\frac{\sigma_{2M+1}}{\sigma_{2M-1}}\right)\\ \; & =\left(\frac{\sigma_{2M-1}+\sigma_{2M}}{\sigma_{2M-3}+\sigma_{2M-2}}-\frac{\sigma_{2M}}{\sigma_{2M-2}}\right)+\left(\frac{\sigma_{2M+1}}{\sigma_{2M-1}}-\frac{\sigma_{2M+1}+\sigma_{2M+2}}{\sigma_{2M-1}+\sigma_{2M}}\right)\\ \; & =\frac{\sigma_{2M}}{\sigma_{2M-2}}\left(\frac{\sigma_{2M-1}/\sigma_{2M}+1}{\sigma_{2M-3}/\sigma_{2M-2}+1}-1\right)\\ \; & +\frac{\sigma_{2M+1}}{\sigma_{2M-1}}\left(1-\frac{1+\sigma_{2M+2}/\sigma_{2M+1}}{1+\sigma_{2M}/\sigma_{2M-1}}\right)\\ \; & \geq 0. \end{array}$$

The other three equations in (A2) can be proved similarly.
